# One-Stop Outpatient Management of Accessory Auricle in Children with Titanium Clip

**DOI:** 10.1155/2014/780394

**Published:** 2014-04-01

**Authors:** Phui Yee Wong, Tereze Laing, Catherine Milroy

**Affiliations:** Department of Plastic and Reconstructive Surgery, St George's Hospital, Blackshaw Road, Tooting, London SW17 0QT, UK

## Abstract

*Introduction*. Literature on ideal management of accessory auricles is limited. Traditionally, accessory auricles are managed by paediatricians with suture ligation at the base of the accessory auricle to induce ischaemic necrosis (Mehmi et al, 2007). This method can be associated with complications and poor cosmesis thus leading to the vogue of surgical excision ( Frieden et al, 1995; Sebben, 1989). We present our experience in managing these lesions in children with the application of a titanium clip in a one-stop outpatient setting. * Methods*. Data was collected retrospectively through review of patient records and telephone questionnaire identifying outcomes from the parents' perspective. * Results*. Of 42 patients, 24 (57.1%) responded. Eleven (26.2%) underwent surgical excision, 6 (14.3%) had no intervention, and 1 (2.4%) was not contactable. All parents were happy with the outcome and would recommend this management to other parents. Twenty-three (96%) had no complications apart from a tiny residual nubbin, which was considered cosmetically acceptable. One child had a residual nubbin that grew in size requiring surgical excision at later stage. * Conclusion*. Management of accessory auricles by the application of a titanium clip in one-stop outpatient setting is safe, simple, quick, and well tolerated with no need for admission, anaesthesia, or followup due to the low complication rate.

## 1. Introduction

The auricle is developed from the fusion of 6 auricular hillocks from the first 2 pharyngeal arches. The process starts around six weeks of gestation and auricular development is usually completed by week twelve. The auricle is initially formed at the base of the neck, but it migrates to the normal adult ear location as the mandible develops by week 20 of gestation, [1].

**Figure 1:**
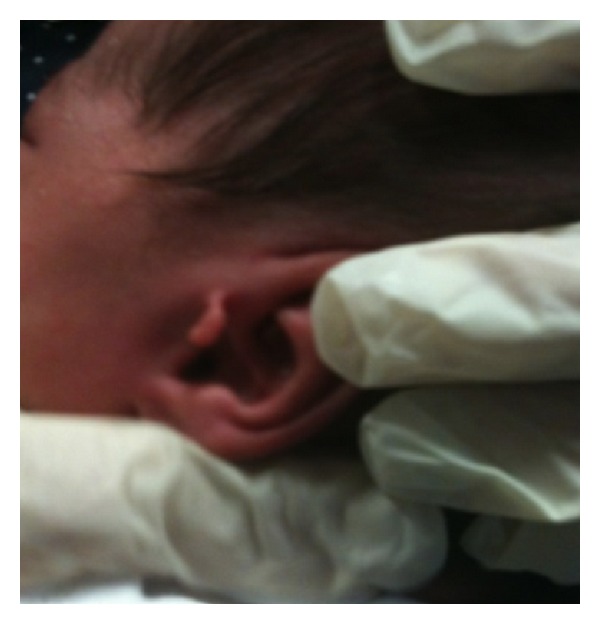
Left accessory auricle.

**Figure 2:**
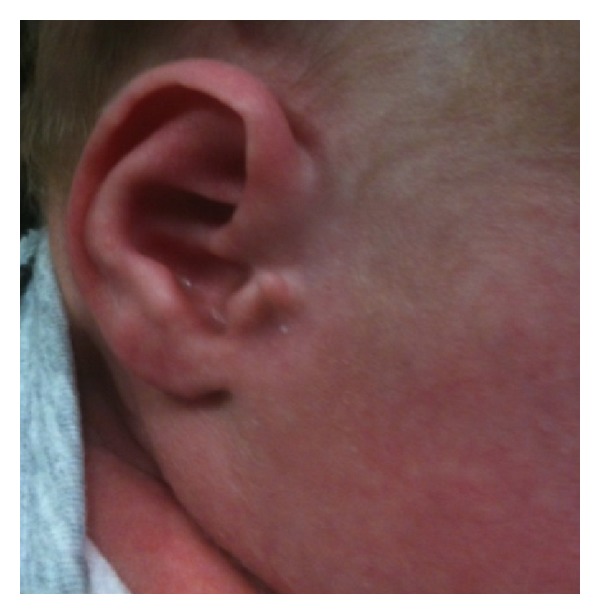
Right accessory auricle.

The accessory auricle is a developmental anomaly resulting from the persistence of a supernumerary auricular hillock, [1]. Accessory auricles can be found in 1.5% of the population [2]. They usually occur as a small elevation of skin with cutaneous appendages with or without a cartilaginous core anterior to the tragus (Figures 1 and 2). Accessory auricles often occur in isolation, but they can occur as part of a syndrome such as Goldenhar or Treacher-Collins syndrome.

Despite being one of the most commonly occurring congenital malformations, there are very few reports in the literature on the management of accessory auricles. Traditionally, these lesions were treated by suture ligation at the base of the accessory auricle to induce ischaemic necrosis [3]. However, This method can be associated with complications and poor cosmesis thus leading to the vogue of surgical excision [4, 5]. Stewart et al. described surgical excision of accessory auricles under local anaesthetic in infants under 2 weeks old. This technique has yielded good cosmetic results with high parental satisfaction, [6]. It can be performed in infants who are referred early, with the appropriate swaddling techniques, feeding, and local anaesthesia in infants up to 3 months old. However, this method usually requires an assistant, a sterile environment, and surgical instruments.

In St George's Hospital, we manage the majority of accessory auricles by application of titanium clip using, the Covidien Premium Surgiclip II in an outpatient setting with no anaesthesia [7]. In the one-stop clinic, we combine initial consultation and management in one appointment. The suitability of the accessory auricle for clip application is determined using the following criteria:

1. there must be no palpable cartilage in the core of the accessory auricle;
2. the pedicle of the lesion must be narrower than the titanium clip;
3. the child should be less than 3 months old.

After assessment for suitability, the options of titanium clip, surgical excision, or no intervention are presented to the parents. The details of each option are explained and informed consent is obtained if the parents are happy to proceed with clip ligation.

**Figure 3:**
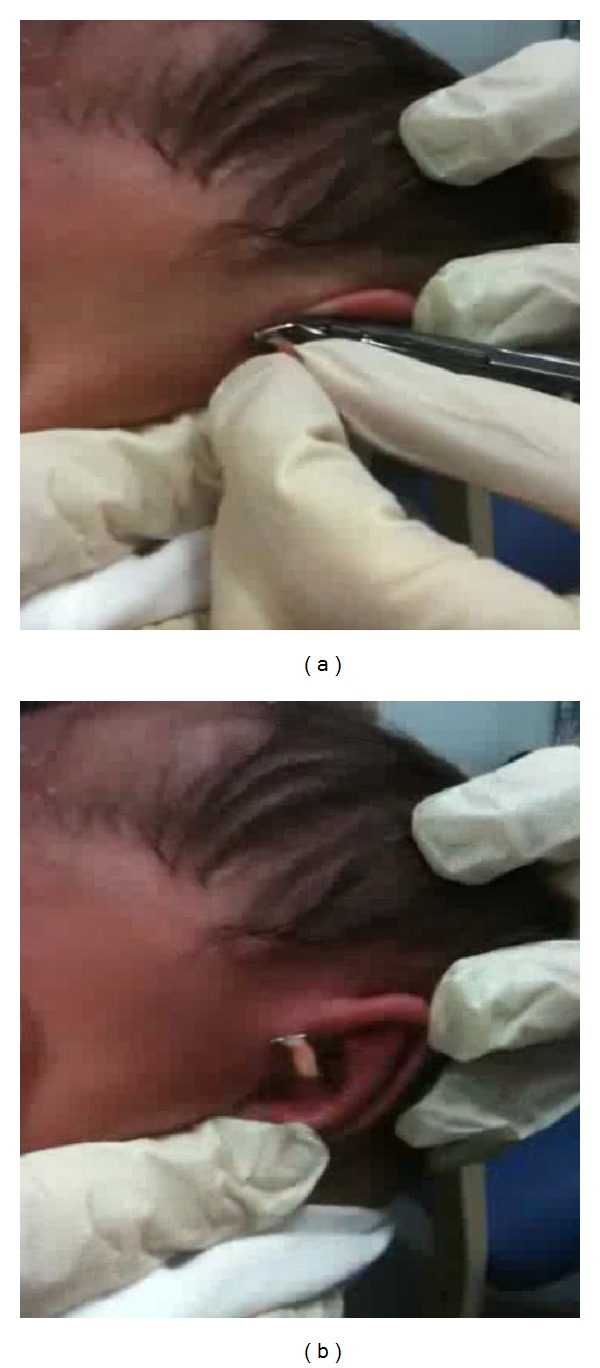
Application of titanium clip at the base of accessory auricle.

The child is wrapped in a blanket and held by the nurse specialist. The preauricular region is cleaned with an alcohol wipe. The accessory auricle is tented slightly and the titanium clip is applied flushed to the skin to avoid a residual nubbin developing in future (Figure 3). Immediately after the clip application, the child is breast- or bottle-fed by parents as a mode of comforting. It is explained to the parents that the clip and the accessory auricle can take from a few days to a couple weeks to necrose and fall off. The parents are also advised to be vigilant for signs of infection such as erythema, swelling, skin breakdown, fever, and poor feeding. A contact number is given in case of any concerns. The child is then discharged from clinic without further followup.

## 2. Methods

We undertook a retrospective study to review outcomes and complications of titanium clip ligation of accessory auricles in infants from parents' perspective. Forty-two infants referred to us between August 2009 and October 2010 were included in the study. Data were collected through review of patients' medical records and a telephone survey to parents using a questionnaire proforma (Section 3). The telephone survey was performed at least 21 months after intervention.

## 3. Questionnaire Proforma

### 3.1. Management of Accessory Auricles with Titanium Clips Telephone Survey Questionnaire Proforma

 Patient's name:
 Date of birth:
 Hospital No:
 Gender:
 Age:
 Telephone no:
 Age/ Date of referral:
 Method of referral: Neonatal unit/ Paediatrics/ GP/ Obstetrics/ Self
 Age/Date when seen in clinic:
 Site of accessory auricle: Left/Right/Bilateral
 Number of days tag fell off after clipped:
 Follow up:
 What were parental concerns before starting treatment?:
 Pain / scar/ cosmetic defects/ infection/ recurrence
 Were the treatment options explained to parents before initiating the treatment?
 Was the patient in any distress after receiving treatment?
 How long did it last for?
  How were they comforted? Breast feeding/ bottle feeding/ analgesia
  Any complications after titanium clip application?
  - Infection
  - Bleeding
  - Visible residual nubbin or scar
  - Need for surgical excision
 Were they happy with the outcome?
 Would they recommend this option to other parents?
 Any further comments?

## 4. Results

Between August 2009 and October 2010, 42 babies were referred to us directly from general practitioners, paediatricians, or obstetricians through a direct referral pathway in the South West London region. Of the 42 babies, 25 (59.5%) had titanium clips applied, 11 (26.2%) underwent surgical excision, and 6 (14.2%) had no intervention. We contacted 41 of 42 parents, and one family was not contactable.

Of the 24 patients who underwent titanium clip application, 13 were male and the remaining were female. Mean age of the patients was 31 days old ranging between 2 days to 3 months old. Seventeen (71%) patients had accessory auricles on the left side and 7 (29%) on the right side. Eighteen (75%) patients had a single accessory auricle and the remaining 6 (25%) had 2 or more. The mean time for the accessory auricle to fall off was 6 days (range 4 to 14 days).

Twenty-three (96%) patients had no complications aside from 11 (46%) patients who reported a residual nubbins, which was barely noticeable and cosmetically acceptable according to parents. There was also no apparent pain, tenderness, or increased sensitivity at the site of the residual nubbin. Only 1 (4%) patient had a residual nubbin, which grew in size and required surgical excision at later stage.

All the infants were fed after the procedure as a method of comforting. Answers relating to the level of discomfort experienced by the infants during and after clip application showed that 10 (42%) cried for a few minutes and resumed feeding normally, 10 (42%) cried for only seconds, and 4 (16%) did not demonstrate any signs of distress or discomfort. There were no reports of infant distress or discomfort at home while waiting for the clipped accessory auricle to fall off.

All parents were pleased with the outcome. All agreed the method was acceptable to them and concurred that having the condition dealt with in a one-stop outpatient setting was convenient as they could go home immediately with no requirement for followup. All parents declared they would recommend this method to others.

## 5. Discussion

The accessory auricle is a congenital malformation of minor significance that rarely presents a problem for the patient or clinician. However, for a minority of parents or patients, it is of sufficient size to cause cosmetic concerns.

When considering removal, it is important to distinguish between lesions that contain a significant cartilaginous component and lesions that do not as failure to remove a protuberant cartilaginous segment may lead to chondrodermatitis at a later date. A number of methods of removal have been described. Suture ligation, when performed correctly, can be as successful as other methods. However, an assistant is required to hold the pedicle so the surgeon's hands are free to tie the knot. Placement of the suture in the correct position at the base of the pedicle can sometimes be difficult especially when the patient is awake which may result in less satisfactory cosmetic outcome. In general, suture ligation has been replaced by surgical removal with successful outcomes reported. Surgical excision requires some form of anaesthesia. It can be done up to 3 to 4 months of age with correct swaddling techniques, feed and wrap techniques, and local anaesthesia but usually requires an assistant and the correct equipment to be available. It is often useful to have diathermy available in the event of excessive bleeding.

The method of titanium clip application in the outpatient clinic is a simple, effective, less time-consuming, and less costly (Table 1) method of managing a large number of these lesions. Although we are aware that we are not the first to use this technique, it has been previously underreported in the literature. Our study demonstrates parent satisfaction and minimal infant distress using this procedure. We have succeeded in avoiding potential complications and reducing departmental costs of surgery in these young infants.

It is important to stress that the technique is not applicable to all cases of accessory auricle. The clip will not successfully traverse a broad pedicle or one that contains cartilage. If cartilage is present, the cartilage can be palpated between the fingertips in the pedicle. These are indications for surgical excision.

In our survey, 46% of parents reported a residual nubbin, which was barely noticeable and cosmetically satisfactory to them. It is quite likely that this would not be an issue following surgical excision. However, we would argue that there would in either case be a scar and that the small disadvantage of a persisting nubbin is outweighed by the many advantages of the clip application procedure.

## 6. Conclusion

In conclusion, we have demonstrated a good overall experience of the use of titanium clip application for the management of accessory auricles with no cartilage core in infants under 3 months old. This is an acceptable method with high parental satisfaction; it is quick and easy to apply with no need for anaesthesia thus allowing patients to go home immediately after the clinic appointment.

## Figures and Tables

**Table 1:** Cost comparison between managing accessory auricle in an outpatient setting using titanium clip and surgical excision as a day case procedure in St. George's Hospital, London, in 2010.

	Outpatient clinic	Day case procedure
Total cost	*£*213.27 (including cost of the Covidien Premium Surgiclip at *£*68.27)	*£*1,305.00

Cost difference	*£*1,091.73	

## Products Used

Covidien Premium Surgiclip II Medium 9.75”.
